# Non-tuberculous mycobacterial pulmonary disease: Awareness survey of front-desk healthcare workers in Northern Tanzania

**DOI:** 10.1371/journal.pgph.0000741

**Published:** 2023-01-20

**Authors:** Togolani Maya, Erick Komba, Gloria Mensah, Nicholaus Mnyambwa, Basra Doulla, Sayoki Mfinanga, Kennedy Addo, Rudovick Kazwala

**Affiliations:** 1 Department of Veterinary Medicine and Public Health, College of Veterinary Medicine and Biomedical Sciences, Sokoine University of Agriculture, Morogoro, Tanzania; 2 Central Tuberculosis Reference Laboratory, National Tuberculosis and Leprosy Program, Dar es Salaam, Tanzania; 3 Department of Bacteriology, Noguchi Memorial Institute for Medical Research, University of Ghana, Accra, Ghana; 4 National Institute for Medical Research, Muhimbili Research Centre, Dar es Salaam, Tanzania; Amsterdam Institute for Global Health and Development, NETHERLANDS

## Abstract

Over the past decade, there have been increasing reports of non-tuberculous mycobacteria (NTM) species being implicated in tuberculosis (TB) treatment failure or misdiagnosed as TB. Inadequate awareness of NTM pulmonary disease among healthcare workers (HCWs) may contribute to a low index of suspicion for patients presenting to their hospitals. In this study, we assessed the awareness of NTM pulmonary disease (NTM-PD) among front desk HCWs in Northern Tanzania. A cross-sectional descriptive survey was carried out among front desk HCWs in four administrative regions of Northern Tanzania. A standardized questionnaire was administered to consented participants from four clusters; clinicians, laboratory scientists, nurses, and pharmacists serving TB patients from Regional and District Health Facilities. Each participant was asked a set of questions, scored and the total score for each participant was determined. An awareness score was used to measure the level of awareness. The average score for all participants was estimated including the 95% confidence interval (CI). The overall awareness score was 24.1%, 95% CI 22.0–26.2%. History of training, experience in TB care, level of health facilities, age group, and setting were found to be statistically associated with the level of awareness of study participants. More than two-thirds (67%) of participants believe that pulmonary NTM and TB are clinically similar and 60% are not aware that AFB Microscopy cannot distinguish between the two. Only 13% of participants could mention at least one risk factor for NTM pulmonary disease. The level of awareness of NTM pulmonary disease was poor among HCWs in the surveyed TB clinics. National TB Programs are advised to include a topic on NTM in various on-job TB training packages for HCWs.

## Introduction

Non-tuberculous mycobacteria (NTM) are mycobacteria species not assigned to *Mycobacterium tuberculosis* complex (MTBC) nor *Mycobacterium leprae*. They are also known as atypical mycobacteria, environmental mycobacteria, opportunistic mycobacteria, or mycobacteria other than tuberculosis (MOTT). These bacteria are ubiquitous in the environment and can be isolated from soil and water including sanitation (chlorine-treated) water [1]. Humans acquire pulmonary NTM infections mainly through inhalation of water droplets from contaminated sources like showers. There is no strong evidence of human-to-human transmission. Bryant et al. and Ruis et al. reported evidence of human-to-human transmission among patients admitted to hospital and among patients with cystic fibrosis respectively [2, 3], but the epidemiological analytical studies have not supported such an evidence [4]. On the other hand, the distribution of NTM varies globally depending on the environmental, microbial, ecological, climatic, and weather characteristics of a place [5]. MTBC causes tuberculosis (TB) and *Mycobacterium leprae* causes leprosy while NTM pulmonary infections can lead to either normal lung colonization or pulmonary disease with TB-like symptoms. This condition is known as NTM pulmonary disease (NTM-PD) [6]. NTM species have frequently been isolated from patients with TB signs [7–10]. As for MTBC, NTMs are also AFB positive on the microscope, Xpert MTB/Rif discriminates and cannot identify these bacteria [11]. Diagnosis of NTMs mainly relies on clinical signs, radiological features, and microbiological assessment of presumptive patients [12].

Although clinical pathogenicity of NTM is not yet well established, the organisms have been associated with a variety of diseases from localized infections to disseminated diseases such as acute or chronic respiratory diseases, lymphadenitis, sinusitis, skin and soft tissue infection [12]. However, NTMs predominantly present as a chronic pulmonary disease [13]. People with other underlying diseases such as Acquired Immuno-deficiency Syndrome (AIDS) and chronic lung diseases such as Cystic Fibrosis (CF) are more prone to the disease. Studies carried out in Kilimanjaro and Arusha Tanzania discovered that NTMs can cause septicemia and lymphadenitis [14, 15]. NTM species have frequently been isolated from patients with TB signs [7–9]. For this reason, more rigorous studies are warranted to assess the pathogenesis of various mycobacterial species like avium and abscessus complex [16–18].

The prevalence and number of NTM species causing disease in humans have been reported to increase over the recent decades. In the USA for example, the prevalence of NTM was found to be high among women and the elderly (65 years and above). Between 2008 and 2015, the prevalence of NTM increased by at least 10% in 39 states [19]. A systematic review and meta-analysis of 37 studies on pulmonary NTM in the South-Saharan countries revealed a prevalence of 7.5% [20]. This includes saprophytes and the emerging species that cause diseases in humans and animals. A study conducted in 2013 reported a total of 140 NTM species [21], while Meier-Kolthoff [22] lists more than 200 NTM species. In Tanzania, two studies carried out in Tanga Region (Tanzania) reported 9.7% [7] and 8.1% [8] of presumptive TB patients were infected with NTM. Despite a significantly high proportion of NTM cases among TB presumptive cases, NTMs have never been considered reportable in programmatic control measures. Early detection of NTM cases through a culture of at least two sputa specimens taken on separate days and correct management ultimately improves treatment success among patients with the NTM-PD [23].

Control of pulmonary NTM disease can be achieved partly through raising awareness of NTM among healthcare workers (HCWs) and thus suspiciousness of patients presenting in their hospitals [19, 24]. A high suspicious index on presumptive TB cases and cases under treatment helps in ruling out NTM and proper management of cases [25]. This study aimed to assess awareness of pulmonary NTM disease among HCWs in TB clinics in Northern Tanzania. The generated information should contribute to proper prevention measures, detection, and management of cases thus reducing incident rate, and treatment failures among misdiagnosed TB patients, and eventually improving the health of people.

## Materials and methods

### Ethics statement

This study protocol was approved by the Sokoine University of Agriculture Institutional Research Review Board, and the Institutional Research Ethical Committee of the National Institute for Medical Research (NIMR) (NIMR/HQ/R.8a/Vol. IX/3245). Gateway permission was obtained from the Medical officer in-charge of each hospital visited. This study obtained a written informed consent from all study participants. Study identification numbers were used instead of subjects’ names to ensure that the identity of each participant remains anonymous. Data obtained were kept in a computer with protected access using a password, and hard copies were kept in a lockable cabinet.

### Study design and setting

**Fig 1:**
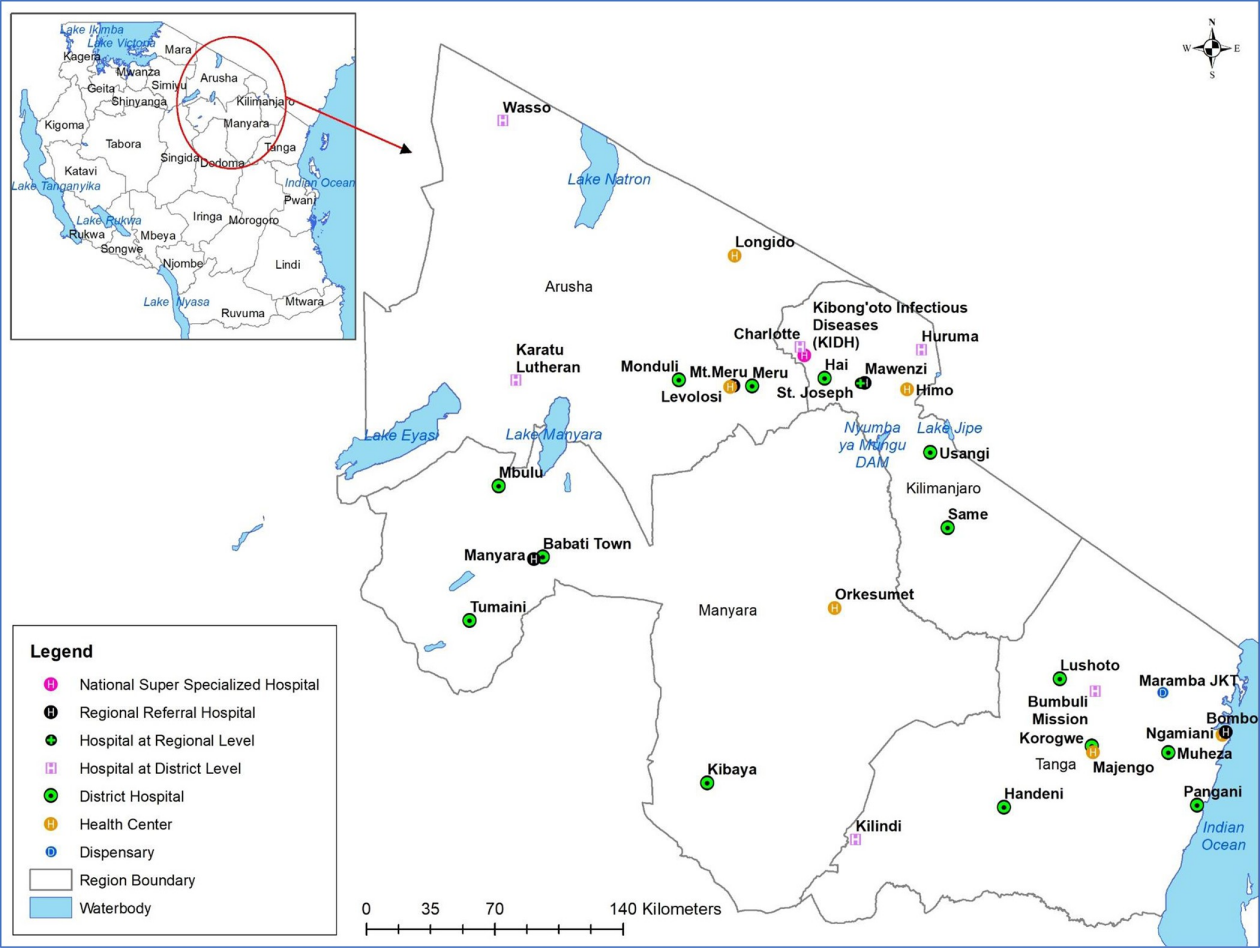
Study site. The map was generated by authors using administrative boundaries shapefile obtained from the Tanzania National Bureau of Statistics.

This was a descriptive cross-sectional survey conducted in Health Facilities located in Northern Tanzania (Tanga, Arusha, Manyara, and Kilimanjaro) between November 2019 to February 2020 (Fig 1). This survey was designed based on the WHO guideline for developing knowledge, attitude, and practice surveys [26]. These regions are among the top ten high TB burden regions in Tanzania [27–32]. It was intended to reach all Regional and District Hospitals.

### Study population and sampling procedure

Health care workers involved in TB care from TB clinics of the selected hospitals formed the study population for this survey. From the four selected regions, we included all Reginal and District Hospitals, a total of 33 Health Facilities (HFs) all with active TB clinics. For each facility, at least one representative from four clusters of TB front-desk healthcare workers namely clinicians, nurses, laboratory technologists, and pharmacists was conveniently selected for the interview. Thus, we estimated to recruit 132 participants (four participants per facility) in this survey. However, due to logistical constraints, we were not able to reach four facilities. Study participants from each cluster were then purposively selected based on their availability during survey visits. Participants who are not involved directly in the diagnosis and management of pulmonary TB were excluded. Only participants who provided written consent were interviewed.

### Questionnaire administration

We conducted face-to-face interviews using a structured questionnaire on the demographic characteristics of the study participants, their general awareness, and awareness on transmission, diagnosis, and management of NTM infections (S1 Questionnaire). The questionnaire was developed based on information gathered from various research findings including multi-center and systematic reviews on NTM, authors’ knowledge, and experience in the field and validated by experts in mycobacteriology. A pre-test was conducted by interviewing ten participants to validate the questionnaire tool. The questionnaire was then revised to rephrase questions that had multiple interpretations, to ensure that they are specific, use simple language to minimize biases as per standard requirements [26]. While twenty-eight questions carrying 32 responses were specific to NTM others were assessing general awareness in the field. Most of the responses were categorical except for age and awareness scores. It took 30 to 45 minutes to administer the questionnaire to each participant.

### Data management and statistical analysis

Collected data were encoded into 2010 Microsoft Excel® spreadsheets (S1 Data). Scoring was done for each participant based on the questions that were specific to the assessment of NTM awareness. Data cleaning and some basic descriptive statistics were done in Microsoft Excel. The data set was then imported to R Studio version 1.2.5033 for further analysis. HCWs levels of awareness were assessed in terms of percentage score from a total of questions asked of which the answers were correct. An unpaired t-test procedure was used to estimate the mean of awareness between males and females HCWs. All variations with a *p-*value less than 0.05 were considered statistically significant.

### Results interpretation

This study used among others “awareness score” as the main outcome variable. Participants were considered aware of a particular research item on the study questionnaire when they gave a correct answer. Each correct answer was scored “one” except for items that required a participant to provide more than one response in which a score of “one” was given when a participant was able to mention only one and a score of “two” was awarded when a participant was able to mention more than one correct response. Each participant was scored out of the total score i.e. 32 and multiplied by 100 to a get percentage score. Scores were ranked as poor, fair, and good as indicated in Table 1 [33]. Percentage awareness of participants was also evaluated based on individual research items like the ability to mention any other name for NTM and any species.

**Table 1:** How score-based awareness was ranked.

S/N	Score ranking (%)	Level of awareness
01	0–49	Poor/Unsatisfactory
02	50–74	Fair/Satisfactory/Moderate
03	75–100	Good/High/Excellent

## Study results

### Study setting analyses

A total of 29 (88%) Regional and District Hospitals were visited out of 33 available in the region. Of these HFs one (3%) was a National TB Reference Hospital, two (7%) were Regional Referral Hospitals (RRHs), and 26 (90%) were District Hospitals (DHs). Of the district hospitals, 13 were public-owned, six were faith-based HFs also called Designated District Hospitals (DDHs) and seven were Healthcare Centres (HCs) for Districts that didn’t have established district hospitals. Tanga Region had the highest number of HFs (11, 38%), while Arusha had the lowest (five, 17%). Four HFs could not be visited due to various reasons, among them being in-accessibility during the study period which resulted from heavy rains (Ngorongoro and Bumbuli District Hospitals). Others were Mawenzi RHH and Mount Meru RRH, the reason being unaffordable research fees.

### Participants’ demographic characteristics

**Fig 2:**
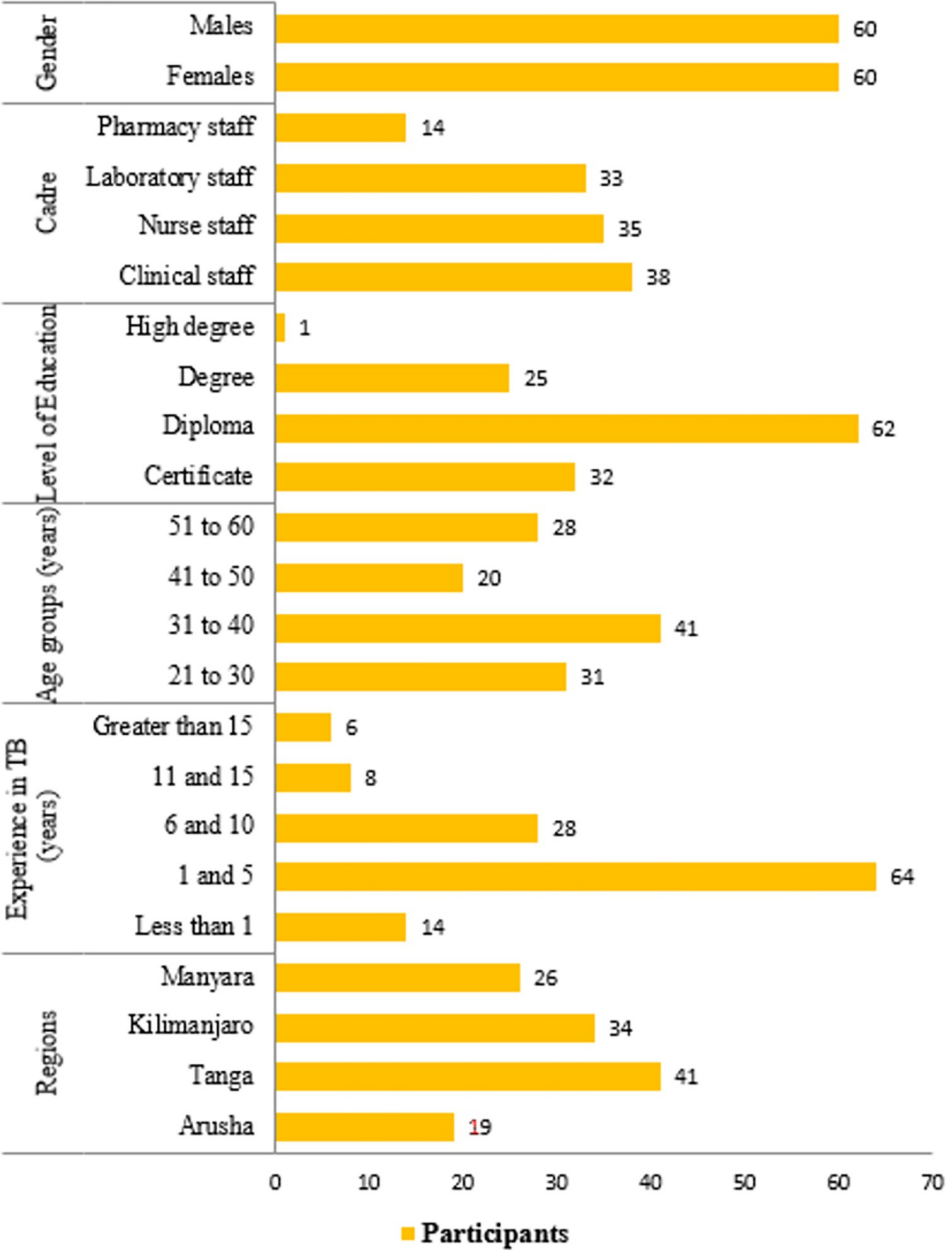
Demographic characteristics of the participants.

**Fig 3:**
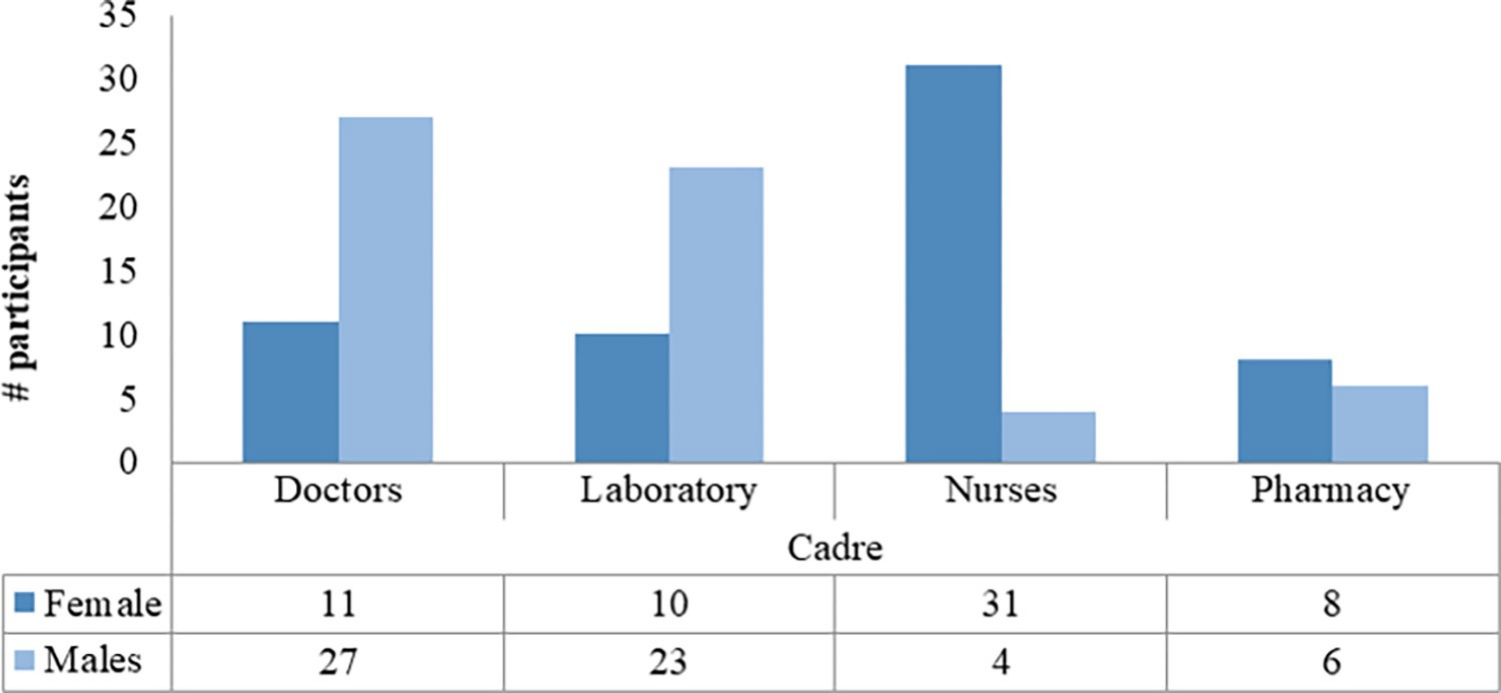
Gender variation for each cadre.

**Fig 4:**
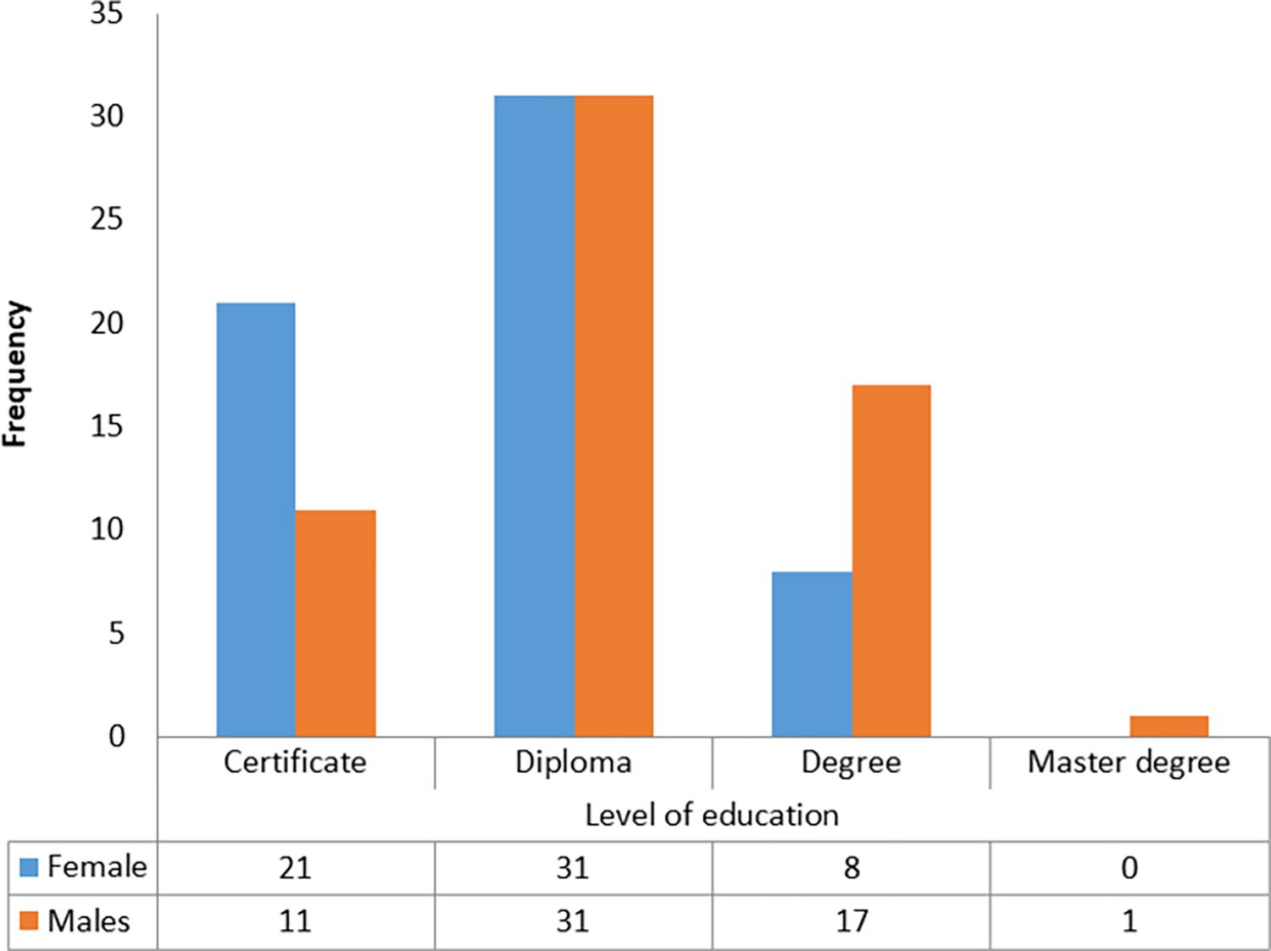
Distribution of participants by education levels.

**Fig 5:**
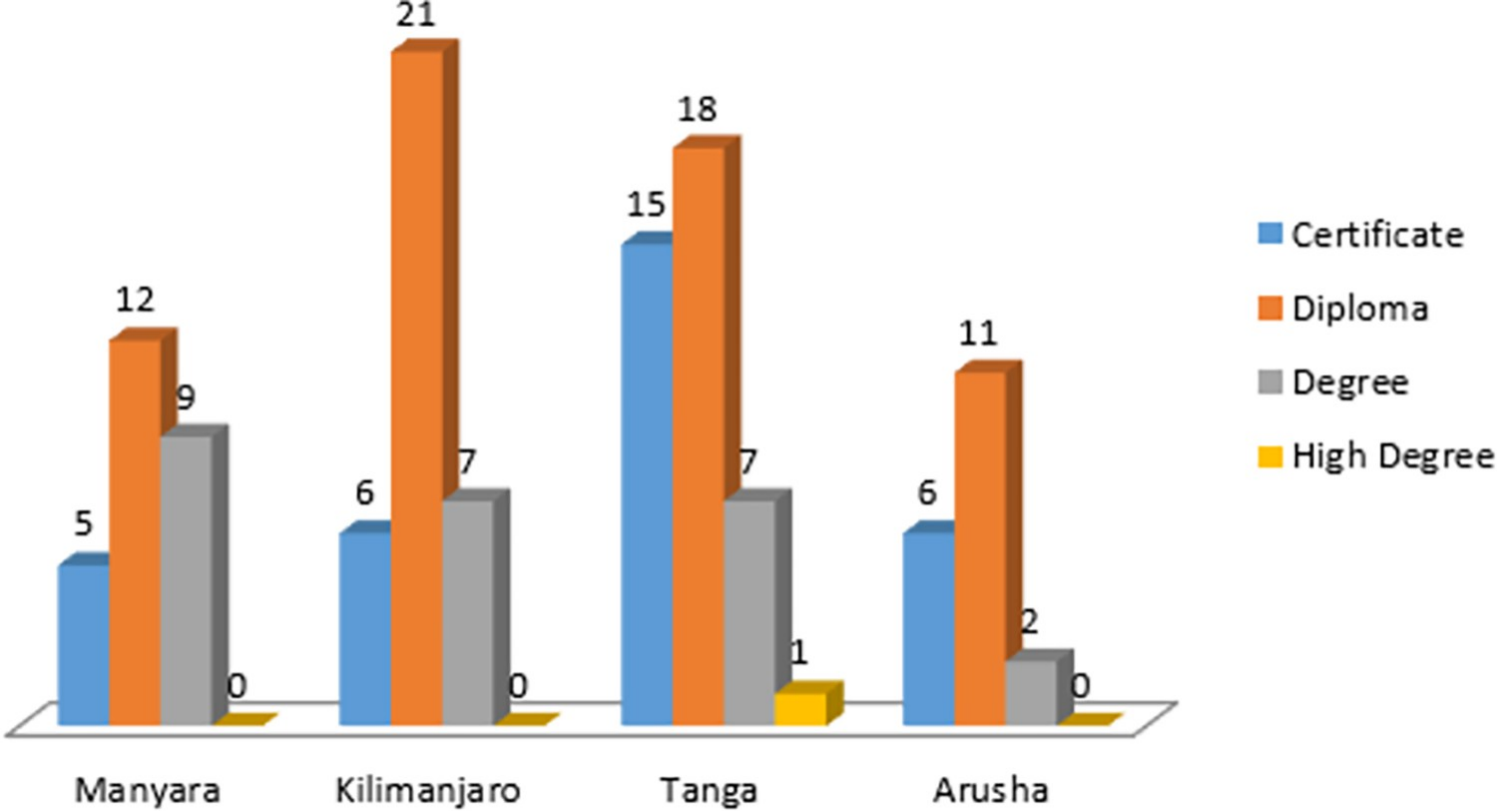
Distribution of participants by level of education in the four regions.

A total of 120 participants were interviewed with an average age of 40 years, 95% CI 38–42 years with a range between 24 to 60 years. It was also noted that most of the participants had a work experience of between 1 and 5 years. While the majority of the participants with younger ages (below 41) were males, the majority of participants with old ages were females. Fig 2 indicates variation in the numbers of respondents among various demographic characteristics. Males and females were coincidentally represented in equal proportion. Most female participants had lower education levels compared to males. Table 2 summarises the numbers of participants per type of HF visited. Males constituted a high proportion of all cadres except nurses of which the number of females was by far higher than that of males (Fig 3). Figs 4 and 5 display the distribution of different cadres of participants based on gender and region.

**Table 2:** Number of study participants for each type of health facility.

Type of Health Facility	Female	Male	Total (%)
NTRH	3	1	4 (3)
RRH	3	6	9 (8)
DH	27	28	55 (46)
DDH	16	13	29 (24)
HC	11	12	23 (19)
**Total**	**60**	**60**	**120**

### Pulmonary NTM disease awareness score results

The mean score for awareness of pulmonary NTM was 24.1%, 95% CI 22.0–26.2%; the highest was 61% and the lowest was 3%. Only five (4%) of all participants had a moderate level of awareness (Table 1) on pulmonary NTM while all the remaining had a low level of awareness. Table 3 summarizes mean awareness scores, 95% CIs, and p-values that measure significance of any variations between the outcome variable and the explanatory variables. Female participants had an average awareness score of 22%, 95% CI 20–24% while among males was 26%, 95% CI 24–28% as displayed in Table 3.

**Table 3:** NTM awareness score among study participants with different attributes.

Explanatory variables	Variability	Mean Awareness Score (%)	95% CI	*p-*value
Gender	Men	26	24–28	0.32
	Women	22	20–24	
Age group (years)	21–30	26	24–28	2.20e-16
	31–40	26	24–28	
	41–50	23	21–25	
	51–60	21	19–23	
Experience in Health Care	Less than 1	13	NA	0.97
	1–5	28	26–30	
	6–10	22	20–24	
	11–15	18	16–20	
	Greater than 15	23	21–25	
Experience in TB care [years]	Less than 1	20	19–21	5.4e-05
	1–5	26	24–28	
	6–10	24	22–26	
	11–15	18	16–20	
	Greater than 15	22	21–23	
Education Level	Certificate	20	18–22	0.85
	Diploma	24	22–26	
	Degree	29	27–31	
	Higher degree	28	NA	
Region	Tanga	25	23–27	0.04
	Arusha	18	17–19	
	Manyara	24	22–26	
	Kilimanjaro	26	24–26	
Cadre	Pharmacist	22	19.5–24.5	0.73
	Nurse	21	19–23	
	Clinician	26	24–28	
	Laboratory personnel	26	24–28	
NTM training	Yes	44	1.25	3.7e-11
	No	24	22–26	
Type of HF	NRH	46	43–49	0.00021
	RRH	33	30–36	
	DH	22	20–24	
	HC	23	21–25	

**Fig 6:**
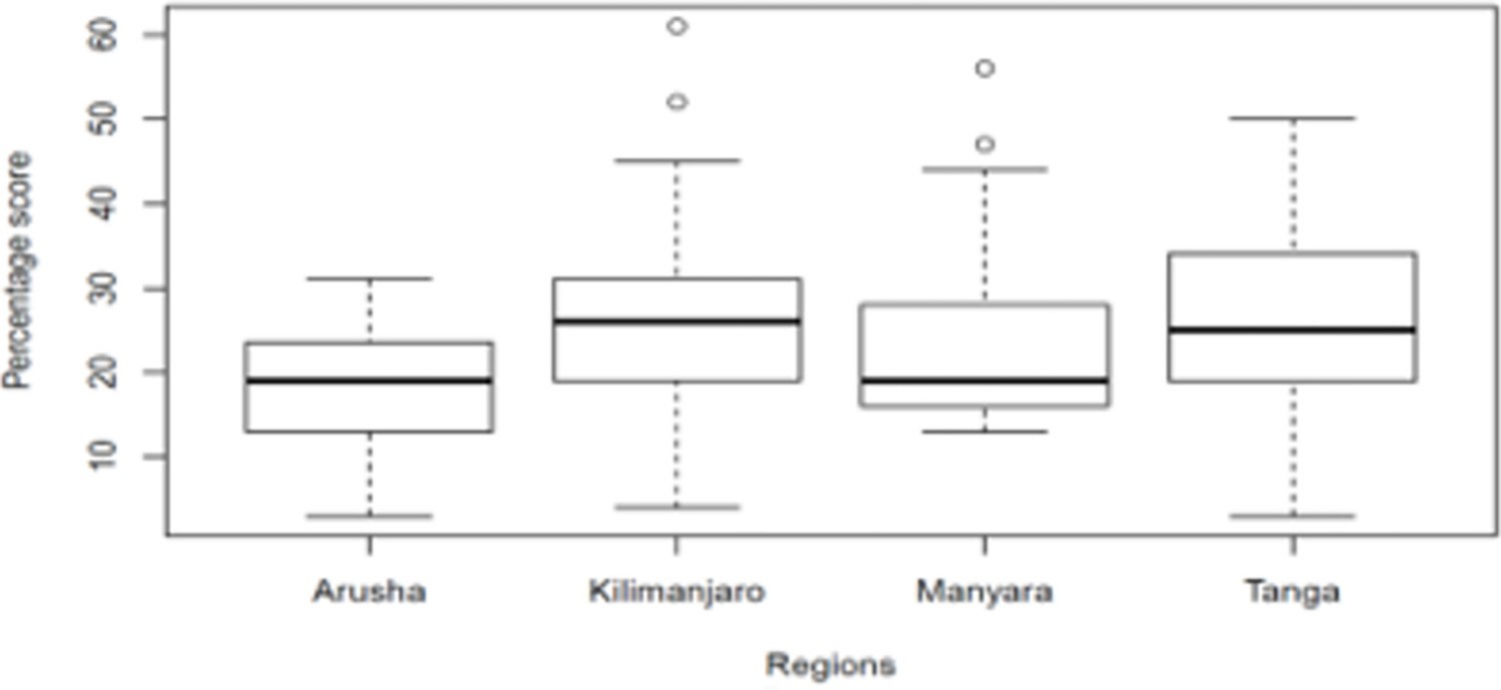
Boxplot of mean pulmonary NTM awareness scores per region.

Kilimanjaro Region had the highest average awareness score of 26%, 95% CI 24–28% followed by Tanga at 25%, 95% CI 23–27% and Manyara at 24%, 95% CI 24–26% while Arusha had the lowest mean awareness score of 18%, 95% CI 17–19% see Fig 6.

Of the 120 participants, only three (3%) had attended a training where NTM was a topic for discussion. It was also found that 79 (66%) of participants were aware that not all mycobacteria species can cause TB. Only 48 (40%) participants reported having ever heard the term non-tuberculous or mycobacteria other than tuberculosis in the whole period of their career. Although only 19 (16%) of participants could correctly define NTM, 77 (64%) believed that pulmonary NTM infection is clinically similar to TB. Thirty-three (28%) of all the interviewed participants were aware that not all NTM can cause diseases in humans. Only 12 (10%) and 12 (12%) participants could mention any other name and species of NTM. Table 4 shows responses to other questions that were asked during this survey.

**Table 4:** Participants’ responses to the survey questions.

Category	Item	Responses	Counts	%
General awareness questions	Ever trained on NTM	Yes	3	2
		No	117	97
	NTM are freely available in water and soil	TRUE	18	15
		FALSE	12	10
		No response	90	75
	Ever heard about NTM/MOTT before	Yes	48	40
		No	72	60
	Able to define NTM	Yes	19	16
		No	101	84
	All NTM can cause diseases in human	TRUE	42	35
		FALSE	33	28
		Not Sure	45	38
	Able to mention any other name for NTM	Able	12	10
		Unable	108	90
Diagnosis of pulmonary NTM	Pulmonary NTM and TB are clinically different	Yes	38	33
		No	77	67
	NTM presents as i] Pulmonary ii] Extra–pulmonary iii] Both	Pulmonary	22	19
		Extra–Pulmonary	28	24
		Both	67	57
	Ability to identify risk factors for NTM infection	Able	72	60
		Unable	48	40
	Microscope cannot distinguish between MTBC and NTM	TRUE	48	40
		FALSE	33	28
		Not Sure	39	32
	GeneXpert can detect both NTM and MTBC	TRUE	51	42
		FALSE	32	27
		Not Sure	37	31
	Which of these is the gold standard test for NTM	Culture	49	41
		GeneXpert	29	24
		Microscopy	7	7
		Not Sure	34	28
	The DNA test is the definitive for NTM	TRUE	44	37
		FALSE	12	10
		Not Sure	64	53
Transmission of NTM	NTM found in soil and water	TRUE	18	15
		FALSE	22	18
		Not Sure	80	67
	Mention Pulmonary NTM risk factor	Able to mention any	15	13
		Can’t mention any	105	87
	Pulmonary NTM is acquired through inhalation	Yes	84	70
		No	36	30
	Can be transmitted from one person to another	TRUE	52	53
		FALSE	19	19
		Not Sure	27	28
Treatment of NTM	All pulmonary infecting NTM species can be treated with TB drugs	TRUE	39	33
		FALSE	29	24
		Not Sure	52	43
	All NTM species can be treated with the same drug regimen	TRUE	15	13
		FALSE	46	38
		Not sure	59	49
	Ability to mention any of the drug recommended for NTM	Mention at least one	13	12
		Couldn’t mention any	98	88
	How long (months) extended treatment takes after NTM case converts to culture negative	3	3	3
		6	27	22
		12	1	1
		24	1	1
		Not sure	85	71

## Discussion

Since pulmonary NTM infections present with signs similar to TB, competence among HCWs in TB clinics will improve the management of cases of this emerging public health threat. The low level of NTM awareness observed in this study supports the recommendations for building awareness among HCWs on the disease especially in TB endemic countries [34]. This is important because the management of pulmonary NTM differs significantly from that of TB. Otherwise, it takes a high suspicious index among HCWs for diagnosis of patients, treatment, and transmission prevention.

The most important finding in this study was the poor awareness of pulmonary NTM infections among HCWs in TB clinics (Table 3). Only four percent had been found to have moderate awareness of pulmonary NTM infections, being a low proportion compared to that found in a similar study on TB awareness in Uganda in which authors reported 62% of the HCWs being aware [35].

The current study found a significant association between awareness and the following factors; experience in TB care, history of training on NTM, age group, type of HF, and administrative region (Table 3). Although there was a variation in mean scores between gender, cadre, level of education, and experience in health care services provision, they were not significantly associated with the level of awareness of NTM.

The observed association of awareness with experience in TB care suggests a difference in proper management of NTM cases between juniors in TB care versus seniors. This calls for interventions like on-job, training, and mentorship of junior HCWs on NTM infections. However, this should consider the contradicting fact that staff with younger age were more aware of NTM compared to elders (Table 3). In addition, the high level of awareness of NTM of staff from RRH compared to DH and HC suggests that an NTM case has a greater chance of proper management at high-level facilities compared to lower levels. This supports the referral system that currently exists in Tanzania. Similarly, NTM infected persons in Kilimanjaro was found to have advantages in receiving proper medical care when compared with a similar patient from the Arusha region which had a lower average score. From a scaled-up similar survey, the information generated will be important in the prioritization of limited resources.

Participants in the age groups of 21 to 30 years and 31 to 40 years had the highest score compared to those in higher age groups. This correlation coincided with the finding that participants with lesser experience on the job i.e. one to five years and six to 10 years (26% 95% CI 24–27 and 24 95% CI 22–26 respectively) had higher awareness scores respectively. This suggests that fresh HCWs in lower age groups and experience had an advantage due to recent graduation from colleges where NTM has been part of most of the current curricula. The history of on-job training of which NTM was a topic of discussion had a strong association with the high level of awareness of NTM. This finding is worth noting and thus formalization of an NTM module in the current TB training packages for HCWs is highly recommended. It will create awareness among practitioners on NTM as a differential diagnosis in situations of TB treatment failures and that not every treatment failure is a result of *Mycobacterium tuberculosis* complex drug resistance [36]. It will also promote the inclusion of NTM as a differential diagnosis in Xpert MTB/Rif tested TB-negative patients who present with symptoms of atypical TB [11]. Although clinicians had a higher mean score than nurses and pharmacists, this study could not find a significant difference in the level of awareness between the cadres of HCWs studied.

A significant number of HCWs (70%) reported that NTMs are acquired mainly through inhalation which is right but only 19% were aware that there is not enough evidence of person-to-person transmission [37]. Despite evidence of NTM being acquired directly from the environment, limited findings support the transmission of *M*. *abscessus* between patients with Cystic Fibrosis [2]. These bacteria are ubiquitous in water and soil, but only 18 (15%) of HCWs were aware of this disease condition. Very few participants were able to mention any risk factor for pulmonary NTM disease but a significant number could report a risk factor of NTM-PD from a list of factors on a multiple-choice question. A good understanding seems to have been influenced by their experience that most lung infectious diseases result from similar risk factors.

The finding that more than two-thirds of respondents were aware that NTM-PD presents with clinical signs similar to TB indicates a strength in the studied population. Most participants were aware that NTM-PD cannot be differentiated from TB clinical features. This suggests the need for laboratory involvement in the confirmation of the disease. Although less than half (40%) of participants were aware that Microscope cannot differentiate NTM from TB and 42% believed that Xpert MTB/Rif can detect NTM [11]. This shows that clinicians have been expecting NTM cases to be reported from laboratories having these test techniques which is contrary to the truth. In addition, less than half of HCWs interviewed were aware that culture is the gold standard test for NTM infections and that PCR test is the definitive test for diagnosis of NTM infection. This shows that clinicians had inadequate knowledge about the diagnosis of NTM and hence a low suspicion index for patients presumed with the disease condition.

Awareness of drugs and regimens recommended for treatment is crucial for the proper management of pulmonary NTM infections. This study found a poor level of awareness among HCWs of drugs and/or regimens for the treatment of pulmonary NTM. Only two in 10 HCWs could mention at least one of the drugs recommended for pulmonary NTM infections. Although MAC and MABC cannot respond to most ant-TB drugs, some NTM species like *Mycobacteria kansasii* respond to Rifampicin, Isoniazid, and Ethambutol. Most participants were not aware that not all NTM species cause pulmonary infections and can be treated with some anti-TB drugs [23, 38, 39]. While MAC responds to macrolides in addition to rifampicin and Ethambutol, MABC responds to macrolides in addition to aminoglycosides and other antibiotics [40].

### Limitations

The questionnaire used needs more validation steps like pilot tests with a larger number of respondents. This is necessary to explore and include comprehensive response options and to minimize leading and confusing questions. For this study, this might have to some extent affected the levels of reliability and validity of data generated hence the inferential statistical measures. Furthermore, the lack of previous studies done on the topic has limited the level of validity of the findings [41]. However, our study shed light on the awareness of NTM among HCWs that warrant further studies and interventions.

## Conclusions and recommendations

The level of awareness of NTM pulmonary disease among health care workers in TB clinics was generally unsatisfactory to be able to diagnose, treat and prevent the disease. The findings call for the inclusion of an NTM component in the TB training package for Healthcare workers. This study provides a baseline for further studies on the topic in the future.

## Supporting information

S1 Data Ntmpd survey dataset. (XLSX)

S1 Questionnaire Survey questionnaire. (DOCX)
