# Supplementary material for: Non-tuberculous mycobacterial pulmonary disease: Awareness survey of front-desk healthcare workers in Northern Tanzania
Source: PLOS Glob Public Health. 2023 Jan 20;3(1):e0000741. doi: 10.1371/journal.pgph.0000741 (PMC10021751; doi:10.1371/journal.pgph.0000741)
Supplement: S1 Questionnaire — (DOCX) [file pgph.0000741.s002.docx]

**Appendix I: Survey Questionnaire: Front desk health care workers’ awareness of nontuberculous mycobacterial pulmonary diseases**

**Interviewee Particulars:**

| Interviewee ID |  | Age |  |
| --- | --- | --- | --- |
| Gender |  | Designation |  |
| Name of TB Clinic |  | District |  |
| Region |  | Phone contact |  |
| Date of interview |  | Name of interviewer |  |

1. What is your highest level of education?
2. Basic/Technical certificate
3. Diploma
4. Bachelor Degree
5. Master Degree
6. PhD
7. Post-Doctoral
8. For how long have you been working as a Health Worker?
9. Less than one year
10. One to 5 years
11. Six to 10 years
12. 11 to 15 years
13. More than 15 years
14. For how long have you (ever) been working in TB Clinic?
    1. Less than one year
    2. One to 5 years
    3. Six to 10 years
    4. 11 to 15 years
    5. More than 15 years
15. Mention a name of bacteria that cause TB; ……………………
16. Do you know what any tests used to detect TB?
    1. No b) Yes
17. If yes, mention all that you know; ……………………………
18. Do you know what tests are used to detect TB in your facility?
19. No b) Yes
20. If yes, mention all that you know; ……………………………..
21. Are you aware of the treatment regimen/drugs for TB in your facility?
22. No b) Yes
23. If yes, mentionall drugs that you know;………………………
24. Are you aware of TBdrug resistance testing in your health facility
    1. No b) Yes
25. Have you ever been involved in sending (referral) of sputum sample for TBdrug resistance testing?
26. Nob) Yes
27. All Mycobacteria species cause TB.
28. True b) False c) Not sure
29. Have you ever heard of Nontuberculous Mycobacteria (NTM) in connection with TB?
30. No b)Yes
31. If yes what are nontuberculous mycobacteria? ………………………………
32. Can pulmonary Nontuberculous Mycobacteria pulmonary infection be differentially diagnosed from pulmonary TB by clinical presentation?
    1. No b) Yes
33. All NontuberculousMycobacteria cause disease in human.
    1. True b) False c) Not sure
34. Mention any other name(s) for NontuberculousMycobacteria; ………………………
35. Mention any species of Nontuberculous mycobacteria that you know; ………………………
36. All Nontuberculous Mycobacterium species can be treated with same drug regimen.
    1. True b)False c)Not sure
37. Mention any drug or group of drugs that are recommended for treatment of pulmonary Nontuberculous Mycobacteria; ………………………………
38. Have you ever diagnosed or been involved in diagnosis or treatment of a patient with nontuberculous mycobacteria infection?
    1. No b) Yes
39. Mention the diagnostic test that was used; …………………………..
40. How long was the treatment course (in months); …………………………
41. How long (in moths)extended treatment takes after culture conversion to “Culture Negative” before NTM patient can be declared treated.
    1. Three b) Six c)12 d)24 e) Not sure
42. Acid Fast Bacilli smear microscopy test cannot distinguish between Mycobacteria that cause TB and NontuberculousMycobacteria.
    1. True b)False c)Not sure
43. Genexpert can detect bothmycobacteria that causeTB and nontuberculous mycobacteria.
    1. True b) False c) Not sure
44. The “gold standard” Laboratory test for diagnosis of nontuberculous mycobacteriais
45. Smear microscopy
46. GeneXpert
47. Culture
48. Not sure
49. Definitive diagnosis of nontuberculous mycobacteria species requires molecular (DNA test) techniques
50. True b) False c) Not sure
51. Do you know any risk factors for nontuberculous mycobacterial infection?
    1. No b) Yes
52. If yes, mention any risk factors you know; …………...………………………
53. Which of these people are most prone to nontuberculous mycobacterial infections?
54. HIV infected persons
55. Patients with Cystic Fibrosis
56. Immunocompromised individuals
57. Old age
58. All the above
59. Nontuberculous mycobacteria infections present as
60. Extra-pulmonary b) Pulmonary c) Both
61. All nontuberculous mycobacterial pulmonary infections are treated with the regular first line TB drugs.
62. True b) False c) Not sure
63. Nontuberculous mycobacteria can be transmitted from one person to another
    1. True b) False c) Not sure
64. Nontuberculous mycobacteria pulmonary infections infection are acquired though
65. Inhalation
66. Ingestion
67. Contact
68. All the above
69. Have you ever attended training (workshop) where nontuberculous mycobacterial infection was a topic for discussion?
70. No b) Yes
71. If yes, where and when, and who organized it? a) …………………………… b) ……………………….
72. What did you learn in the training? ……………………………..
73. Would you be interested to attend a workshop on nontuberculous mycobacterial pulmonary infections?
74. No b) Yes c) Not sure
75. Give a reason for your choice; ……………………………………
76. Nontuberculous mycobacteria can easily be found in water and soil.
    1. True b) False c) Not sure

----------------------------------------- END ------------------------------------------------
