# Supplementary material for: Non-tuberculous mycobacterial pulmonary disease: Awareness survey of front-desk healthcare workers in Northern Tanzania
Source: PLOS Glob Public Health. 2023 Jan 20;3(1):e0000741. doi: 10.1371/journal.pgph.0000741 (PMC10021751; doi:10.1371/journal.pgph.0000741)
Supplement: Attachment — Submitted filename: Response to Reviewers.docx [file pgph.0000741.s003.docx]

Dear Academic Editor, Plos Global Public Health

Thank you for providing us with an opportunity to submit a revised draft of our manuscript titled “Non-tuberculous mycobacterial pulmonary disease: Awareness survey of front-desk healthcare workers in Northern Tanzania”. We appreciate the time and effort that you and the reviewers have dedicated to providing your valuable feedback on our manuscript. We are grateful to the reviewers for their insightful comments on our paper. We have been able to incorporate changes to reflect most of the suggestions provided. We have highlighted the changes within the manuscript.

Here is a point-by-point response to the comments and concerns.

Journal Requirements:

***Comment 1***

*1. Please provide additional details regarding participant consent. In the ethics statement in the Methods, please ensure that you have specified (1) whether consent was informed and (2) what type you obtained (for instance, written or verbal, and if verbal, how it was documented and witnessed).*

**Response 1**

A statement**, “**This study provided a written informed consent to each of the potential participants” has been added under the **Ethical clearance and HCW enrolment** section of the manuscript.

***Comment 2***

*2. Please include additional information regarding the survey or questionnaire used in the study and ensure that you have provided sufficient details that others could replicate the analyses. For instance, if you developed a questionnaire as part of this study and it is not under a copyright more restrictive than CC-BY, please include a copy, in both the original language and English, as Supporting Information*.

**Response 2**

The “**Questionnaire administration and data collection**” section of the manuscript is rephrased to provide sufficient details. A copy of the questionnaire used is included as supporting information**.**

***Comment 3***

*3. Please update the 'Competing Interests' statement, including any COIs declared by your co-authors. If you have no competing interests to declare, please state "The authors have declared that no competing interests exist"*

**Response 3**

The competing interest statement is updated to read "The authors have declared that no competing interests exist"

***Comment 4***

*4. Please amend your detailed Financial Disclosure statement. This is published with the article. It must therefore be completed in full sentences and contain the exact wording you wish to be published.*

*- State the initials, alongside each funding source, of each author to receive each grant.
- State what role the funders took in the study. If the funders had no role in your study, please state: “The funders had no role in study design, data collection and analysis, decision to publish, or preparation of the manuscript*.”

Response 4

The Financial Disclosure Statement should read “This work was funded by the DELTAS Africa Initiative (Afrique One-ASPIRE/DEL-15-008). Afrique One-ASPIRE is funded by a consortium of donors including the African Academy of Sciences (AAS)Alliance for Accelerating Excellence in Science in Africa (AESA), the New Partnership for Africa’s Development Planning and Coordinating (NEPAD) Agency, the Wellcome Trust (107753/A/15/Z), and the UK government. The sponsors had no involvement in the study design, data collection, or in the interpretation of the data, and the views expressed in this piece of research are those of the authors and not necessarily those of Afrique One-ASPIRE”.

***Comment 5***

*5. In the online submission form, you indicated that "No legal or ethical restrictions to sharing of data for this study". All PLOS journals now require all data underlying the findings described in their manuscript to be freely available to other researchers, either 1. In a public repository, 2. Within the manuscript itself, or 3. Uploaded as supplementary information.
This policy applies to all data except where public deposition would breach compliance with the protocol approved by your research ethics board. If your data cannot be made publicly available for ethical or legal reasons (e.g., public availability would compromise patient privacy), please explain your reasons by return email and your exemption request will be escalated to the editor for approval. Your exemption request will be handled independently and will not hold up the peer review process, but will need to be resolved should your manuscript be accepted for publication. One of the Editorial team will then be in touch if there are any issues.*

**Response 5**

This is agreed upon and thank you. The data will be shared with the journal during the resubmission of the manuscript as part of supporting information

***Comment 6***

*6. The resolution of Figures are very low and somewhat difficult to read. It is important that our Editors and Peer Reviewers are able to read all parts of a submission. Please replace these figures with higher resolution copies.*

**Response 6**

The figures are replaced with ones with higher resolution

***Comment 7***

*7. Please remove the embedded figures from the manuscript file and upload it as tif or eps format.*

**Response 7**

The figures are uploaded separately in tif format

**Reviewers' Comments to the Author**
Reviewer's Responses to Questions

*1. Does this manuscript meet PLOS Global Public Health’s* [*publication criteria*](https://journals.plos.org/globalpublichealth/s/criteria-for-publication)*? Is the manuscript technically sound, and do the data support the conclusions? The manuscript must describe methodologically and ethically rigorous research with conclusions that are appropriately drawn based on the data presented.*

*Reviewer #1: Yes*

**Response R1**

We are thankful for this compliment

*Reviewer #2: No*

**Response R2**

We have revised the entire manuscript to ensure it meets *PLOS Global Public Health’s* [*publication criteria*](https://journals.plos.org/globalpublichealth/s/criteria-for-publication)*.* This includes revising the methodology among other sections and correcting mentioned grammatical errors. We have rephrased the proposed paragraphs to make the information easier to replicate.

*2. Has the statistical analysis been performed appropriately and rigorously?*

*Reviewer #1: Yes*

**Response R1**

We are thankful for this compliment

*Reviewer #2: I don't know*

**Response R2**

We tried to report the level of awareness using awareness score. We have analysed the level of association and different factors such as cadre, work experience, region, history of training, education qualification, etc. to be able to recommend necessary approaches to improve the level of awareness.

***3. Have the authors made all data underlying the findings in their manuscript fully available (please refer to the Data Availability Statement at the start of the manuscript PDF file)?

The*** [***PLOS Data policy***](https://journals.plos.org/globalpublichealth/s/data-availability) ***requires authors to make all data underlying the findings described in their manuscript fully available without restriction, with rare exception. The data should be provided as part of the manuscript or its supporting information, or deposited to a public repository. For example, in addition to summary statistics, the data points behind means, medians and variance measures should be available. If there are restrictions on publicly sharing data—e.g. participant privacy or use of data from a third party—those must be specified.***

***Reviewer #1: Yes***

**Response R2**

We are grateful for this complement

*Reviewer #2: Yes*

**Response R2**

We are grateful for this complement

*4. Is the manuscript presented in an intelligible fashion and written in standard English?

PLOS Global Public Health does not copyedit accepted manuscripts, so the language in submitted articles must be clear, correct, and unambiguous. Any typographical or grammatical errors should be corrected at revision, so please note any specific errors here.*

*Reviewer #1: Yes*

**Response R1**

We are grateful for this complement

Reviewer #2: Yes

**Response R2**

We are grateful for this complement

*5. Review Comments to the Author

Please use the space provided to explain your answers to the questions above. You may also include additional comments for the author, including concerns about dual publication, research ethics, or publication ethics. (Please upload your review as an attachment if it exceeds 20,000 characters)*

*Reviewer #1: No additional comments. Congratulations to the authors. The manuscript contains valuable information that can be useful for other healthcare workers in the future. Looking forward to the publication of this article.*

**Response R1**

We are grateful for the complement

*Reviewer #2: Line 37: Change “consented” to “consenting”.

Line 66: The numbered citation ‘3’ seems to be a typographical error.

Line 67: An adversative conjunction is missing to relate the two contrasting clauses of the sentence.

Line 73: The use of “and” does not seem to be appropriate.

Section on Study population and sampling procedure: The minimum required sample size targeted has not been indicated, including its basis.

Line 123: Spell out “HF”, the acronym being referred to for the first time in the document.

Line 124: The auxiliary verb “were” is missing before the word “interviewed” (but not before “consented”, which is an intransitive verb).

Line 133: The comma must follow the word “staff”, not “only”.

Section on Questionnaire administration and data collection: Provide more details on the validation process done.

Line 137: Insert “a” before “structured”.

Line 139: Insert “were” before “based”.

Line 146: Insert “the” before “variables”.

Section on Data management and statistical analysis: If p-values were generated, then the study could not just have employed descriptive statistics. Moreover, the statistical tests used (descriptive and inferential) have to be indicated.

Lines 149-150: The sentence can be simplified to “**Collected data were encoded into 2010 Microsoft Excel® spreadsheets.”

Line 153: Capitalize ‘E’ in “excel”.

Line 155: Suggest rephrasing “According to this study all variations…” to “All study results…”.

Section on Results interpretation: Provide the rationale for Table 1. Cross-reference to the comment on the questionnaire validation process.

Line 178: Delete “A” before “Study identification numbers”.

Lines 186, 188: Spell out “RRH”, “DH”, and “HC”; acronyms being referred to for the first time in the document.

Lines 188-189: Suggest rephrasing “The highest number of HFs (11, 38%) was from Tanga region” to “The Tanga region had the highest number of participating HFs (11, 38%)”.

Line 190: Insert a comma after “reasons”.

Line 216: This must be Table 2; we have Table 1 in Line 170.

Table 1 (or 2, as corrected), and Figures 1-4: The information in these table and figures can be incorporated into just one master table showing the profile of the participants and their distribution according to sociodemographic variables, cadre, health facility, and region.

Line 233: Replace “An” with “The”.

Section on Pulmonary NTM disease awareness score results: A choice may to be made as to whether descriptive statistics (mean score, standard deviation, range) or inferential statistics (point and interval estimates of the mean score, p-values) would be reported. Also consider the implications for interpretation.

Lines 233-240: If association between the outcome variable and each explanatory variable is to be established, then inferential statistics (hypothesis testing) is needed; cross-reference to the comment on employing only descriptive statistics. Moreover, interesting patterns from Table 3, other than gender, are better described as part of the narrative.

Figure 5: By comparing the awareness scores across the four regions, are the scores generated by participants from each region representative of all the HCWs (at least from the four cadres) of that region?

Line 253: This must be Table 3.

Table 2 (or 3, as corrected): Indicate the statistical test/s used that generated the p-values. Also, instead of reporting the standard deviations, the interval estimates may be more meaningful given that the objective of the table entails the use of inferential statistics.

Lines 255-256: Delete “On the other hand”. Also, kindly indicate the source of the information in this sentence, as it is not part of Table 4.

Line 268: This must be Table 4.

Table 3 (or 4, as corrected): Format can still be improved; suggest text alignment to the top of the cell, and row borders or hanging indentation to delineate between items and/or responses.

Lines 283-288: Move down the sentences starting from “The current study has found…” to separate paragraphs. Moreover, the* *implication of the observed association of awareness with (1) age, (2) experience in TB care, (3) type of HF, and (4) administrative region were not discussed; while the similarity in correlation patterns of age and work experience have been pointed out (lines 299-304), the more practical applications of these information can still be explored to the extent similar to the discussion for training (lines 290-297).

Lines 290-291: Indicate the source of the information in this sentence; cross-reference to comment on lines 255-256.

Discussion section: A discussion of results related to participant responses related to diagnosis of pulmonary NTM—similar to dedicated paragraphs for transmission (lines 308-319) and treatment (lines 321-330)—is missing.

Limitations section: If the validation process for the tool developed is a limitation, describe the nature and extent to which it may have affected the results of the study; cross-reference to comment on questionnaire administration and data collection. Moreover, indicate likely threats to validity that are linked to selection and participant response.

Conclusions and Recommendations section: This section can still be improved and expanded, following comments made on the Results and Discussion sections.*

**Response R2**

Several typographical or grammatical errors have been corrected on the entire manuscript;

Line 66: ‘3’ has been deleted

Line 67: Adversative ‘but’ has been inserted

Section on “Study population and sampling procedure” minimum sample size and selection modality has been indicated

Line 123: “HF” has been spelled out as “Health Facility”

Line 124: The paragraph is rephrased

Line 133: The comma is moved to the position that follows the word staff

Section on Questionnaire administration and data collection: The paragraph is rephrased to accommodate the recommendations

Line 137: ‘a’ is inserted before structured

Line 139: The statement is rephrased

Line 146: ‘the’ is inserted

Lines 149-150: The recommendation has been considered

Section on Data management and statistical analysis: Inferential statistics are now indicated with clarity in the section, Standard deviations have been replaced by 95% CI.

Line 153: ‘e’ in excel is capitalized

Line 155: Statement rephrased to start with ‘All variations...’

Section on Results interpretation: Table one shows how awareness was ranked in this study, the table is cited and supports the findings and conclusion of this study.

Line 178: “A” deleted before “Study”

Lines 186, 188: RRH”, “DH”, and “HC”; acronyms are spelled out as Regional Referral Hospital, District Hospital, and Health Centre respectively

Lines 188-189: “The highest number of HFs (11, 38%) was from Tanga region” is rephrased to “The Tanga region had the highest number of participating HFs (11, 38%)”.

Line 190: A comma is inserted after “reasons”.

Line 216: This table was mislabelled, it is renamed as Table 2.

We think that a lot of pieces of messages would get dropped off if we will try to merge Tables 1 and 2 and Figures 1-4. This will make information intended to be delivered incomplete and difficult to replicate.

Line 233: “An” has been replaced with “The”.

Section on Pulmonary NTM disease awareness score results: Despite using descriptive statistics to summarise characteristics of our data set this study went further to use inferential statistics to determine to what extent the data are generalizable for the population. However, 95% CI has been omitted from line 234 as it is independent of Standard Deviation and relay close message.

Lines 233-240: The idea of using a Table 3 was to summarize a narrative that could be generated from it and provide a single quick view of the number of explanatory variables in a snapshot

Yes, this study analyzed variation of generalized HCWs awareness scores per Region aiming to determine any existence of a difference in the level of awareness between them. Using p-value this specific study supported the difference as indicated on Table 3.

Line 253: The table is Table 3

In table 3 and the entire document, the standard deviations have changed to 95% Confidence Intervals

Lines 255-256: “On the other hand” is deleted. More items and their response rate have been added to the table

Line 268: This Discussion section discusses results from Table 3 and not 4

Table 3 suggestions taken on board, the table is formatted

Lines 283-288: The line is moved down to separate the paragraphs. More discussion has been added on the implication of observed association of awareness with (1) age, (2) experience in TB care, (3) type of HF, and (4) administrative region to explore more practical application of the information.

Lines 290-291: The source of the information is inserted in this sentence

Discussion section: A paragraph dedicated for diagnosis of NTM infection has been added 330-341 similar to transmission (lines 308-319) and treatment (lines 321-330)

Limitations section: This section has been revised to include areas that might have been affected due to the stated limitations

Conclusions and Recommendations section: Referring to the manuscript this section is expanded and improved as recommended.

*6. PLOS authors have the option to publish the peer review history of their article (*[*what does this mean?*](https://journals.plos.org/globalpublichealth/s/editorial-and-peer-review-process#loc-peer-review-history)*). If published, this will include your full peer review and any attached files.*


***Do you want your identity to be public for this peer review?*** *If you choose “no”, your identity will remain anonymous but your review may still be made public.


For information about this choice, including consent withdrawal, please see our* [*Privacy Policy*](https://www.plos.org/privacy-policy)*.*

*Reviewer #1:* ***Yes:*** *Evalyn A. Roxas*

**Response R1**

We are grateful for the complement

*Reviewer #2: No*

**Response R2**

We are grateful for the insightful and constructive comments

We look forward to hearing from you in due time regarding our resubmission and we will be grateful to receive and further address your comments.

Sincerely,

Togolani Godfrey Maya 11/06/2022
