# Supplementary material for: Non-tuberculous mycobacterial pulmonary disease: Awareness survey of front-desk healthcare workers in Northern Tanzania
Source: PLOS Glob Public Health. 2023 Jan 20;3(1):e0000741. doi: 10.1371/journal.pgph.0000741 (PMC10021751; doi:10.1371/journal.pgph.0000741)
Supplement: Attachment — Submitted filename: Response to Reviewers.docx [file pgph.0000741.s004.docx]

Dear Academic Editor, Plos Global Public Health

Thank you for providing us with another opportunity to make further improvement of our submission manuscript titled “Non-tuberculous mycobacterial pulmonary disease: Awareness survey of front-desk healthcare workers in Northern Tanzania”. We appreciate the time and effort that you and the reviewers have dedicated to providing your valuable feedback on our manuscript. We are grateful to the reviewers for their insightful comments on our manuscript. We have been able to incorporate changes to reflect most of the suggestions provided, which have improved the content and appeal of the manuscript. We have highlighted the changes within the manuscript.

Here is a point-by-point response to the comments and concerns.

Journal Requirements:

***Comment 1***

*1. Please review your reference list to ensure that it is complete and correct. If you have cited papers that have been retracted, please include the rationale for doing so in the manuscript text, or remove these references and replace them with relevant current references. Any changes to the reference list should be mentioned in the rebuttal letter that accompanies your revised manuscript. If you need to cite a retracted article, indicate the article’s retracted status in the References list and also include a citation and full reference for the retraction notice.*

**Response 1;**

We revisited the references and made changes whenever deemed relevant. Reference number 20, which was an obsolete citation has been changed to the currently recommended citation.

*Additional Editor Comments (if provided):*

***Comments 2***

*This article is very close to publication.*

**Response 2**

We are grateful for this complement

***Comment 3***

*I would, as Reviewer 2 outlined, adjust the CI and provide clarification to statistical tests used.*

**Response 3**

Inferential statistics, both p-value and CI have been provided to determine the level of association between variables

***Comment 4***

*In the intro and conclusion, I would try to bring some clarity to the NTM transmission, as it is one of the study questions. I would simply state that non-tuberculous mycobacteria is almost entirely from the environment and almost entirely not person-to-person, but could be in rare cases. Subsequent to the papers cited in the intro, there has been work that points to person-to-person transmission, particularly among CF patients.*

*https://www.nature.com/articles/s41564-021-00963-3*

**Response**

We have revised NTM transmission statement to capture uncertainties of transmission between environment to human and human to human with each scenario backed up by the citations evidencing the situation.

***Comment 5***

*I would expand discussion of existing knowledge of NTM epi in Tanzania (possibly could decrease discussion of US NTM). I would explain the what range of NTM found in Tanzania, even just the number of types found to date (ie it's not just MAC and abscessus) https://bmcresnotes.biomedcentral.com/articles/10.1186/s13104-016-1928-3/tables/4*

**Response 5**

The single sentence on NTM in the US in the introduction section provided the global situation of NTM, this then was narrowed down to the Africa and Tanzania context despite the limited number of studies that have so far been conducted on NTM from distribution/epidemiology, burden, species, and impact on public health. Moreover, the discussion section has been devoted to discussing the findings of this NTM Pulmonary Disease (NTM-PD) Awareness Survey of and association of awareness with other categorical variables of which species so far found in Tanzania was not part of it.

[Note: HTML markup is below. Please do not edit.]

Reviewers' comments:

Reviewer's Responses to Questions

Comments to the Author

*1. If the authors have adequately addressed your comments raised in a previous round of review and you feel that this manuscript is now acceptable for publication, you may indicate that here to bypass the “Comments to the Author” section, enter your conflict of interest statement in the “Confidential to Editor” section, and submit your "Accept" recommendation*.

***Comment 1 R1***

*Reviewer #1: All comments have been addressed*

**Response 1 R1**

We are grateful for this compliment

***Comment 1 R2***

*Reviewer #2: (No Response)*

**Response 1 R2**

We are grateful for this comment. We hope this means nothing is apparently needed to be addressed

***Comment 1 R3***

*Reviewer #3: All comments have been addressed*

**Response 1 R3**

We are grateful for this compliment

*2. Does this manuscript meet PLOS Global Public Health’s publication criteria? Is the manuscript technically sound, and do the data support the conclusions? The manuscript must describe methodologically and ethically rigorous research with conclusions that are appropriately drawn based on the data presented.*

***Comment 2 R1***

*Reviewer #1: Yes*

**Response 2 R1**

We are grateful for this compliment

***Comment 2 R2***

*Reviewer #2: Partly*

**Response 2 R2**

We have revised this paper we hope now it fully meet the PLOS Global Public Health’s publication criteria

***Comment 2 R3***

*Reviewer #3: Yes*

**Response 2 R3**

We are grateful for this compliment

*3. Has the statistical analysis been performed appropriately and rigorously?*

***Comment 3 R1***

Reviewer #1: Yes

**Response 3 R1**

We are grateful for this compliment

***Comment 3 R2***

Reviewer #2: I don't know

**Response 3 R2**

***We look foward to address more comments when arise***

***Comment 3 R3***

*Reviewer #3: Yes*

**Response 3 R3**

We are grateful for this compliment

*4. Have the authors made all data underlying the findings in their manuscript fully available (please refer to the Data Availability Statement at the start of the manuscript PDF file)?*

*The PLOS Data policy requires authors to make all data underlying the findings described in their manuscript fully available without restriction, with rare exception. The data should be provided as part of the manuscript or its supporting information, or deposited to a public repository. For example, in addition to summary statistics, the data points behind means, medians and variance measures should be available. If there are restrictions on publicly sharing data—e.g. participant privacy or use of data from a third party—those must be specified.*

***Comment 4 R1***

*Reviewer #1: Yes*

**Response 4 R1**

We are grateful for this compliment

***Comment 4 R2***

*Reviewer #2: No*

**Response 4 R2**

All data underlying findings have been made available and accessible as supporting information

***Comment 4 R3***

Reviewer #3: Yes

**Response 4 R3**

We are grateful for this compliment

*5. Is the manuscript presented in an intelligible fashion and written in standard English?*

*PLOS Global Public Health does not copyedit accepted manuscripts, so the language in submitted articles must be clear, correct, and unambiguous. Any typographical or grammatical errors should be corrected at revision, so please note any specific errors here.*

***Comment 5 R1***

*Reviewer #1: Yes*

**Response 5 R1**

We are grateful for this compliment

***Comment 5 R2***

*Reviewer #2: Yes*

**Response 5 R2**

We are grateful for this compliment

***Comment 5 R3***

Reviewer #3: Yes

**Response 5 R3**

We are grateful for this compliment

*6. Review Comments to the Author*

*Please use the space provided to explain your answers to the questions above. You may also include additional comments for the author, including concerns about dual publication, research ethics, or publication ethics. (Please upload your review as an attachment if it exceeds 20,000 characters)*

***Comment 6 R1***

*Reviewer #1: - Comments from reviewer 2 was addressed appropriately.*

**Response 6 R1**

We are grateful for this compliment

***Comment 6 R1***

*Reviewer #2: The manuscript was significantly improved from the original submission, in terms of content (depth of discussion), grammar, and form. Most of the comments raised in the previous round of review have also been addressed.*

*Still:*

*(1) The specific statistical tests/test criteria that generated the p-values and confidence intervals have yet to be reported.*

**Response 6 R2**

The p-value was calculated from an unpaired test-test procedure. This information is added to the methods section of this manuscript

***Comment 6 R2***

*(2) The 95% CIs are presented in an unconventional way--they appear more like standard deviations, since only one number per measure is shown. Indicate both lower and upper limits of each interval in the tables and narrative.*

**Response 6 R2**

The 95% CIs have been described as +/- on the column title to minimize congestion in the table

***Comment 6 R2***

*(3) A Data Availability Statement, detailing where the data can be accessed, must be categorically stated.*

**Response 6 R2**

The Data Availability Statement has been revised to read as *“*All-important datasets used and/or analyzed in this study are provided in the main text as supplementary materials”

***Comment 6 R2***

*Reviewer #3: The overlapping clinical symptoms due to infection with M. tuberculosis and NTMs often leads to misdiagnosis and hence mistreatment. This study highlights the importance of awareness program among different cadres of health care workers for correct diagnosis and treatment of TB and NTM infections in various pulmonary diseases especially under limited resource settings. Also, authors suggestion to addon-job training for differential diagnosis of NTMs and related awareness to all cadres of HCWs is very important and needs sincere attentions of the policy makers to include this in TB control program.*

*Although authors have adequately addressed the comments of the previous reviewers. I have couple of following minor queries which may be addressed for better clarity.*

*1. Line No 234: Please clarify what is meant here by 'experience between 1 and 5 years'?? what kind of experience is it?*

**Response 5 R2**

This statement was hanging an has been rephrased and now reads “It was also noted that most participants had a work experience of between 1 and 5 years.

***Comment 5 R2***

*2. Line 237: Please provide a range of age for defining Lower and higher ages for males and females respectively*.

**Response 5 R2**

The statement has been rephrased and words lower and higher are replaced with younger and old.

*7. PLOS authors have the option to publish the peer review history of their article (what does this mean?). If published, this will include your full peer review and any attached files.*

*Do you want your identity to be public for this peer review? If you choose “no”, your identity will remain anonymous but your review may still be made public.*

*For information about this choice, including consent withdrawal, please see our Privacy Policy.*

***Comment 7 R1***

*Reviewer #1: No*

**Response 7 R1**

We are grateful for your contribution to improve this manuscript

***Comment 7 R2***

*Reviewer #2: No*

**Response 7 R2**

We are grateful for your contribution to improve this manuscript

***Comment 7 R3***

*Reviewer #3: No*

**Response 7 R3**

**Authors response:** We are grateful for your contribution to improve this manuscript

With kind regards
