# Supplementary material for: Non-tuberculous mycobacterial pulmonary disease: Awareness survey of front-desk healthcare workers in Northern Tanzania
Source: PLOS Glob Public Health. 2023 Jan 20;3(1):e0000741. doi: 10.1371/journal.pgph.0000741 (PMC10021751; doi:10.1371/journal.pgph.0000741)
Supplement: Peer review history

# Peer review: Decision Letter 0

**DOI:** 10.1371/journal.pgph.0000741.r001

**Authors:** Carl Abelardo T. Antonio

2 May 2022

PGPH-D-21-00419

Non-tuberculous Mycobacterial pulmonary disease: Awareness survey of front-desk healthcare workers in Northern Tanzania

PLOS Global Public Health

Dear Dr. Maya,

Thank you for submitting your manuscript to PLOS Global Public Health. After careful consideration, we feel that it has merit but does not fully meet PLOS Global Public Health’s publication criteria as it currently stands. Therefore, we invite you to submit a revised version of the manuscript that addresses the points raised during the review process.

Please submit your revised manuscript by Jun 16 2022 11:59PM. If you will need more time than this to complete your revisions, please reply to this message or contact the journal office at globalpubhealth@plos.org. When you're ready to submit your revision, log on to https://www.editorialmanager.com/pgph/ and select the 'Submissions Needing Revision' folder to locate your manuscript file.

Please include the following items when submitting your revised manuscript:

- A rebuttal letter that responds to each point raised by the editor and reviewer(s). You should upload this letter as a separate file labeled 'Response to Reviewers'.
- A marked-up copy of your manuscript that highlights changes made to the original version. You should upload this as a separate file labeled 'Revised Manuscript with Track Changes'.
- An unmarked version of your revised paper without tracked changes. You should upload this as a separate file labeled 'Manuscript'.

Guidelines for resubmitting your figure files are available below the reviewer comments at the end of this letter.

We look forward to receiving your revised manuscript.

Kind regards,

Carl Abelardo T. Antonio

Academic Editor

PLOS Global Public Health

Journal Requirements:

1. Please provide additional details regarding participant consent. In the ethics statement in the Methods, please ensure that you have specified (1) whether consent was informed and (2) what type you obtained (for instance, written or verbal, and if verbal, how it was documented and witnessed).

2. Please include additional information regarding the survey or questionnaire used in the study and ensure that you have provided sufficient details that others could replicate the analyses. For instance, if you developed a questionnaire as part of this study and it is not under a copyright more restrictive than CC-BY, please include a copy, in both the original language and English, as Supporting Information.

3. Please update the 'Competing Interests' statement, including any COIs declared by your co-authors. If you have no competing interests to declare, please state "The authors have declared that no competing interests exist"

4. Please amend your detailed Financial Disclosure statement. This is published with the article. It must therefore be completed in full sentences and contain the exact wording you wish to be published.

- State the initials, alongside each funding source, of each author to receive each grant.

- State what role the funders took in the study. If the funders had no role in your study, please state: “The funders had no role in study design, data collection and analysis, decision to publish, or preparation of the manuscript.”

5. In the online submission form, you indicated that "No legal or ethical restrictions to sharing of data for this study". All PLOS journals now require all data underlying the findings described in their manuscript to be freely available to other researchers, either 1. In a public repository, 2. Within the manuscript itself, or 3. Uploaded as supplementary information.

This policy applies to all data except where public deposition would breach compliance with the protocol approved by your research ethics board. If your data cannot be made publicly available for ethical or legal reasons (e.g., public availability would compromise patient privacy), please explain your reasons by return email and your exemption request will be escalated to the editor for approval. Your exemption request will be handled independently and will not hold up the peer review process, but will need to be resolved should your manuscript be accepted for publication. One of the Editorial team will then be in touch if there are any issues.

6. The resolution of Figures are very low and somewhat difficult to read. It is important that our Editors and Peer Reviewers are able to read all parts of a submission. Please replace these figures with higher resolution copies.

7. Please remove the embedded figures from the manuscript file and upload it as tif or eps format.

Additional Editor Comments (if provided):

I perused the manuscript and weighed the reviewers' recommendations. After careful consideration, I am inclined to follow the suggestion of the second reviewer, who raised some methodological concerns about the study.

[Note: HTML markup is below. Please do not edit.]

Reviewers' comments:

Reviewer's Responses to Questions

**Comments to the Author**

1. Does this manuscript meet PLOS Global Public Health’s publication criteria? Is the manuscript technically sound, and do the data support the conclusions? The manuscript must describe methodologically and ethically rigorous research with conclusions that are appropriately drawn based on the data presented.

Reviewer #1: Yes

Reviewer #2: No

**********

2. Has the statistical analysis been performed appropriately and rigorously?

Reviewer #1: Yes

Reviewer #2: I don't know

**********

3. Have the authors made all data underlying the findings in their manuscript fully available (please refer to the Data Availability Statement at the start of the manuscript PDF file)?

The PLOS Data policy requires authors to make all data underlying the findings described in their manuscript fully available without restriction, with rare exception. The data should be provided as part of the manuscript or its supporting information, or deposited to a public repository. For example, in addition to summary statistics, the data points behind means, medians and variance measures should be available. If there are restrictions on publicly sharing data—e.g. participant privacy or use of data from a third party—those must be specified.

Reviewer #1: Yes

Reviewer #2: Yes

**********

4. Is the manuscript presented in an intelligible fashion and written in standard English?

PLOS Global Public Health does not copyedit accepted manuscripts, so the language in submitted articles must be clear, correct, and unambiguous. Any typographical or grammatical errors should be corrected at revision, so please note any specific errors here.

Reviewer #1: Yes

Reviewer #2: Yes

**********

5. Review Comments to the Author

Please use the space provided to explain your answers to the questions above. You may also include additional comments for the author, including concerns about dual publication, research ethics, or publication ethics. (Please upload your review as an attachment if it exceeds 20,000 characters)

Reviewer #1: No additional comments. Congratulations to the authors. The manuscript contains valuable information that can be useful for other healthcare workers in the future. Looking forward to the publication of this article.

Reviewer #2: Line 37: Change “consented” to “consenting”.

Line 66: The numbered citation ‘3’ seems to be a typographical error.

Line 67: An adversative conjunction is missing to relate the two contrasting clauses of the sentence.

Line 73: The use of “and” does not seem to be appropriate.

Section on Study population and sampling procedure: The minimum required sample size targeted has not been indicated, including its basis.

Line 123: Spell out “HF”, the acronym being referred to for the first time in the document.

Line 124: The auxiliary verb “were” is missing before the word “interviewed” (but not before “consented”, which is an intransitive verb).

Line 133: The comma must follow the word “staff”, not “only”.

Section on Questionnaire administration and data collection: Provide more details on the validation process done.

Line 137: Insert “a” before “structured”.

Line 139: Insert “were” before “based”.

Line 146: Insert “the” before “variables”.

Section on Data management and statistical analysis: If p-values were generated, then the study could not just have employed descriptive statistics. Moreover, the statistical tests used (descriptive and inferential) have to be indicated.

Lines 149-150: The sentence can be simplified to “Collected data were encoded into 2010 Microsoft Excel® spreadsheets.”

Line 153: Capitalize ‘E’ in “excel”.

Line 155: Suggest rephrasing “According to this study all variations…” to “All study results…”.

Section on Results interpretation: Provide the rationale for Table 1. Cross-reference to the comment on the questionnaire validation process.

Line 178: Delete “A” before “Study identification numbers”.

Lines 186, 188: Spell out “RRH”, “DH”, and “HC”; acronyms being referred to for the first time in the document.

Lines 188-189: Suggest rephrasing “The highest number of HFs (11, 38%) was from Tanga region” to “The Tanga region had the highest number of participating HFs (11, 38%)”.

Line 190: Insert a comma after “reasons”.

Line 216: This must be Table 2; we have Table 1 in Line 170.

Table 1 (or 2, as corrected), and Figures 1-4: The information in these table and figures can be incorporated into just one master table showing the profile of the participants and their distribution according to sociodemographic variables, cadre, health facility, and region.

Line 233: Replace “An” with “The”.

Section on Pulmonary NTM disease awareness score results: A choice may to be made as to whether descriptive statistics (mean score, standard deviation, range) or inferential statistics (point and interval estimates of the mean score, p-values) would be reported. Also consider the implications for interpretation.

Lines 233-240: If association between the outcome variable and each explanatory variable is to be established, then inferential statistics (hypothesis testing) is needed; cross-reference to the comment on employing only descriptive statistics. Moreover, interesting patterns from Table 3, other than gender, are better described as part of the narrative.

Figure 5: By comparing the awareness scores across the four regions, are the scores generated by participants from each region representative of all the HCWs (at least from the four cadres) of that region?

Line 253: This must be Table 3.

Table 2 (or 3, as corrected): Indicate the statistical test/s used that generated the p-values. Also, instead of reporting the standard deviations, the interval estimates may be more meaningful given that the objective of the table entails the use of inferential statistics.

Lines 255-256: Delete “On the other hand”. Also, kindly indicate the source of the information in this sentence, as it is not part of Table 4.

Line 268: This must be Table 4.

Table 3 (or 4, as corrected): Format can still be improved; suggest text alignment to the top of the cell, and row borders or hanging indentation to delineate between items and/or responses.

Lines 283-288: Move down the sentences starting from “The current study has found…” to separate paragraph. Moreover, the implication of the observed association of awareness with (1) age, (2) experience in TB care, (3) type of HF, and (4) administrative region were not discussed; while the similarity in correlation patterns of age and work experience have been pointed out (lines 299-304), the more practical applications of these information can still be explored to the extent similar to the discussion for training (lines 290-297).

Lines 290-291: Indicate the source of the information in this sentence; cross-reference to comment on lines 255-256.

Discussion section: A discussion of results related to participant responses related to diagnosis of pulmonary NTM—similar to dedicated paragraphs for transmission (lines 308-319) and treatment (lines 321-330)—is missing.

Limitations section: If the validation process for the tool developed is a limitation, describe the nature and extent to which it may have affected the results of the study; cross-reference to comment on questionnaire administration and data collection. Moreover, indicate likely threats to validity that are linked to selection and participant response.

Conclusions and Recommendations section: This section can still be improved and expanded, following comments made on the Results and Discussion sections.

**********

6. PLOS authors have the option to publish the peer review history of their article (what does this mean?). If published, this will include your full peer review and any attached files.

**Do you want your identity to be public for this peer review?** If you choose “no”, your identity will remain anonymous but your review may still be made public.

For information about this choice, including consent withdrawal, please see our Privacy Policy.

Reviewer #1: **Yes: **Evalyn A. Roxas

Reviewer #2: No

**********

[NOTE: If reviewer comments were submitted as an attachment file, they will be attached to this email and accessible via the submission site. Please log into your account, locate the manuscript record, and check for the action link "View Attachments". If this link does not appear, there are no attachment files.]

While revising your submission, please upload your figure files to the Preflight Analysis and Conversion Engine (PACE) digital diagnostic tool, https://pacev2.apexcovantage.com/. PACE helps ensure that figures meet PLOS requirements. To use PACE, you must first register as a user. Registration is free. Then, login and navigate to the UPLOAD tab, where you will find detailed instructions on how to use the tool. If you encounter any issues or have any questions when using PACE, please email PLOS at figures@plos.org. Please note that Supporting Information files do not need this step.

# Author comment: Author response to Decision Letter 0

**DOI:** 10.1371/journal.pgph.0000741.r002

23 Jun 2022

Attachment Submitted filename: Response to Reviewers.docx Click here for additional data file.

# Peer review: Decision Letter 1

**DOI:** 10.1371/journal.pgph.0000741.r003

**Authors:** Megan Coffee

3 Aug 2022

PGPH-D-21-00419R1

Non-tuberculous Mycobacterial pulmonary disease: Awareness survey of front-desk healthcare workers in Northern Tanzania

PLOS Global Public Health

Dear Dr. Togolani Godfrey Maya, 

Thank you for submitting your manuscript to PLOS Global Public Health. After careful consideration, we feel that it has merit and is almost ready for publication, but does not fully meet PLOS Global Public Health’s publication criteria as it currently stands. Therefore, we invite you to submit a revised version of the manuscript that addresses the minor points raised during the review process.

Please submit your revised manuscript by Sep 17 2022 11:59PM. If you will need more time than this to complete your revisions, please reply to this message or contact the journal office at globalpubhealth@plos.org. When you're ready to submit your revision, log on to https://www.editorialmanager.com/pgph/ and select the 'Submissions Needing Revision' folder to locate your manuscript file.

Please include the following items when submitting your revised manuscript:

- A rebuttal letter that responds to each point raised by the editor and reviewer(s). You should upload this letter as a separate file labeled 'Response to Reviewers'.
- A marked-up copy of your manuscript that highlights changes made to the original version. You should upload this as a separate file labeled 'Revised Manuscript with Track Changes'.
- An unmarked version of your revised paper without tracked changes. You should upload this as a separate file labeled 'Manuscript'.

Guidelines for resubmitting your figure files are available below the reviewer comments at the end of this letter.

We look forward to receiving your revised manuscript.

Kind regards,

Megan Coffee, MD, PhD

Academic Editor

PLOS Global Public Health

Journal Requirements:

1. Please review your reference list to ensure that it is complete and correct. If you have cited papers that have been retracted, please include the rationale for doing so in the manuscript text, or remove these references and replace them with relevant current references. Any changes to the reference list should be mentioned in the rebuttal letter that accompanies your revised manuscript. If you need to cite a retracted article, indicate the article’s retracted status in the References list and also include a citation and full reference for the retraction notice.

Additional Editor Comments (if provided):

This article is very close to publication.

I would, as Reviewer 2 outlined, adjust the CI and provide clarification to statistical tests used.

In the intro and conclusion, I would try to bring some clarity to the NTM transmission, as it is one of the study questions. I would simply state that non-tuberculous mycobacteria is almost entirely from the environment and almost entirely not person-to-person, but could be in rare cases. Subsequent to the papers cited in the intro, there has been work that points to person-to-person transmission, particularly among CF patients.

https://www.nature.com/articles/s41564-021-00963-3

I would expand discussion of existing knowledge of NTM epi in Tanzania (possibly could decrease discussion of US NTM). I would explain the what range of NTM found in Tanzania, even just the number of types found to date (ie it's not just MAC and abscessus) https://bmcresnotes.biomedcentral.com/articles/10.1186/s13104-016-1928-3/tables/4

[Note: HTML markup is below. Please do not edit.]

Reviewers' comments:

Reviewer's Responses to Questions

**Comments to the Author**

1. If the authors have adequately addressed your comments raised in a previous round of review and you feel that this manuscript is now acceptable for publication, you may indicate that here to bypass the “Comments to the Author” section, enter your conflict of interest statement in the “Confidential to Editor” section, and submit your "Accept" recommendation.

Reviewer #1: All comments have been addressed

Reviewer #2: (No Response)

Reviewer #3: All comments have been addressed

**********

2. Does this manuscript meet PLOS Global Public Health’s publication criteria? Is the manuscript technically sound, and do the data support the conclusions? The manuscript must describe methodologically and ethically rigorous research with conclusions that are appropriately drawn based on the data presented.

Reviewer #1: Yes

Reviewer #2: Partly

Reviewer #3: Yes

**********

3. Has the statistical analysis been performed appropriately and rigorously?

Reviewer #1: Yes

Reviewer #2: I don't know

Reviewer #3: Yes

**********

4. Have the authors made all data underlying the findings in their manuscript fully available (please refer to the Data Availability Statement at the start of the manuscript PDF file)?

The PLOS Data policy requires authors to make all data underlying the findings described in their manuscript fully available without restriction, with rare exception. The data should be provided as part of the manuscript or its supporting information, or deposited to a public repository. For example, in addition to summary statistics, the data points behind means, medians and variance measures should be available. If there are restrictions on publicly sharing data—e.g. participant privacy or use of data from a third party—those must be specified.

Reviewer #1: Yes

Reviewer #2: No

Reviewer #3: Yes

**********

5. Is the manuscript presented in an intelligible fashion and written in standard English?

PLOS Global Public Health does not copyedit accepted manuscripts, so the language in submitted articles must be clear, correct, and unambiguous. Any typographical or grammatical errors should be corrected at revision, so please note any specific errors here.

Reviewer #1: Yes

Reviewer #2: Yes

Reviewer #3: Yes

**********

6. Review Comments to the Author

Please use the space provided to explain your answers to the questions above. You may also include additional comments for the author, including concerns about dual publication, research ethics, or publication ethics. (Please upload your review as an attachment if it exceeds 20,000 characters)

Reviewer #1: - Comments from reviewer 2 was addressed appropriately.

Reviewer #2: The manuscript was significantly improved from the original submission, in terms of content (depth of discussion), grammar, and form. Most of the comments raised in the previous round of review have also been addressed.

Still:

(1) The specific statistical tests/test criteria that generated the p-values and confidence intervals have yet to be reported.

(2) The 95% CIs are presented in an unconventional way--they appear more like standard deviations, since only one number per measure is shown. Indicate both lower and upper limits of each interval in the tables and narrative.

(3) A Data Availability Statement, detailing where the data can be accessed, must be categorically stated.

Reviewer #3: The overlapping clinical symptoms due to infection with M. tuberculosis and NTMs often leads to misdiagnosis and hence mistreatment. This study highlights the importance of awareness program among different cadres of health care workers for correct diagnosis and treatment of TB and NTM infections in various pulmonary diseases especially under limited resource settings. Also authors suggestion to addon-job training for differential diagnosis of NTMs and related awareness to all cadres of HCWs is very important and needs sincere attentions of the policy makers to include this in TB control program.

Although authors have adequately addressed the comments of the previous reviewers. I have couple of following minor queries which may be addressed for better clarity.

1. Line No 234: Please clarify what is meant here by 'experience between 1 and 5 years'?? what kind of experience is it?

2. Line 237: Please provide a range of age for defining Lower and higher ages for males and females respectively.

**********

7. PLOS authors have the option to publish the peer review history of their article (what does this mean?). If published, this will include your full peer review and any attached files.

**Do you want your identity to be public for this peer review?** If you choose “no”, your identity will remain anonymous but your review may still be made public.

For information about this choice, including consent withdrawal, please see our Privacy Policy.

Reviewer #1: No

Reviewer #2: No

Reviewer #3: No

**********

[NOTE: If reviewer comments were submitted as an attachment file, they will be attached to this email and accessible via the submission site. Please log into your account, locate the manuscript record, and check for the action link "View Attachments". If this link does not appear, there are no attachment files.]

While revising your submission, please upload your figure files to the Preflight Analysis and Conversion Engine (PACE) digital diagnostic tool, https://pacev2.apexcovantage.com/. PACE helps ensure that figures meet PLOS requirements. To use PACE, you must first register as a user. Registration is free. Then, login and navigate to the UPLOAD tab, where you will find detailed instructions on how to use the tool. If you encounter any issues or have any questions when using PACE, please email PLOS at figures@plos.org. Please note that Supporting Information files do not need this step.

# Author comment: Author response to Decision Letter 1

**DOI:** 10.1371/journal.pgph.0000741.r004

12 Sep 2022

Attachment Submitted filename: Response to Reviewers.docx Click here for additional data file.

# Peer review: Decision Letter 2

**DOI:** 10.1371/journal.pgph.0000741.r005

**Authors:** Sabine Hermans

17 Nov 2022

PGPH-D-21-00419R2

Non-tuberculous mycobacterial pulmonary disease: Awareness survey of front-desk healthcare workers in Northern Tanzania

PLOS Global Public Health

Dear Dr. Maya,

Please accept our apologies for the protracted nature of this submission and review process. Thank you for your patience. We would like to accept your manuscript; before doing so we would like to ask you to submit a version in which you report your 95% confidence intervals in the way that is customary in this research field (as a range, rather than +/-). We appreciate your concern about congestion of text and tables, however this is preferable to non-customary reporting.

Thank you for submitting your manuscript to PLOS Global Public Health. After careful consideration, we feel that it has merit but does not fully meet PLOS Global Public Health’s publication criteria as it currently stands. Therefore, we invite you to submit a revised version of the manuscript that addresses the points raised during the review process.

==============================

Please see my comments above; there is no need to submit a "response to the reviewers" document.

==============================

Please submit your revised manuscript by Jan 01 2023 11:59PM. If you will need more time than this to complete your revisions, please reply to this message or contact the journal office at globalpubhealth@plos.org. When you're ready to submit your revision, log on to https://www.editorialmanager.com/pgph/ and select the 'Submissions Needing Revision' folder to locate your manuscript file.

Please include the following items when submitting your revised manuscript:

- A marked-up copy of your manuscript that highlights changes made to the original version. You should upload this as a separate file labeled 'Revised Manuscript with Track Changes'.
- An unmarked version of your revised paper without tracked changes. You should upload this as a separate file labeled 'Manuscript'.

Guidelines for resubmitting your figure files are available below the reviewer comments at the end of this letter.

We look forward to receiving your revised manuscript.

Kind regards,

Sabine Hermans

Academic Editor

PLOS Global Public Health

Journal Requirements:

1. Please include additional information regarding the survey or questionnaire used in the study and ensure that you have provided sufficient details that others could replicate the analyses. For instance, if you developed a questionnaire as part of this study and it is not under a copyright more restrictive than CC-BY, please include a copy, in both the original language and English, as Supporting Information.

Please review your reference list to ensure that it is complete and correct. If you have cited papers that have been retracted, please include the rationale for doing so in the manuscript text, or remove these references and replace them with relevant current references. Any changes to the reference list should be mentioned in the rebuttal letter that accompanies your revised manuscript. If you need to cite a retracted article, indicate the article’s retracted status in the References list and also include a citation and full reference for the retraction notice.

Additional Editor Comments (if provided):

[Note: HTML markup is below. Please do not edit.]

Reviewers' comments:

Reviewer's Responses to Questions

**Comments to the Author**

1. If the authors have adequately addressed your comments raised in a previous round of review and you feel that this manuscript is now acceptable for publication, you may indicate that here to bypass the “Comments to the Author” section, enter your conflict of interest statement in the “Confidential to Editor” section, and submit your "Accept" recommendation.

Reviewer #2: (No Response)

Reviewer #3: All comments have been addressed

**********

2. Does this manuscript meet PLOS Global Public Health’s publication criteria? Is the manuscript technically sound, and do the data support the conclusions? The manuscript must describe methodologically and ethically rigorous research with conclusions that are appropriately drawn based on the data presented.

Reviewer #2: Yes

Reviewer #3: Yes

**********

3. Has the statistical analysis been performed appropriately and rigorously?

Reviewer #2: Yes

Reviewer #3: Yes

**********

4. Have the authors made all data underlying the findings in their manuscript fully available (please refer to the Data Availability Statement at the start of the manuscript PDF file)?

The PLOS Data policy requires authors to make all data underlying the findings described in their manuscript fully available without restriction, with rare exception. The data should be provided as part of the manuscript or its supporting information, or deposited to a public repository. For example, in addition to summary statistics, the data points behind means, medians and variance measures should be available. If there are restrictions on publicly sharing data—e.g. participant privacy or use of data from a third party—those must be specified.

Reviewer #2: Yes

Reviewer #3: Yes

**********

5. Is the manuscript presented in an intelligible fashion and written in standard English?

PLOS Global Public Health does not copyedit accepted manuscripts, so the language in submitted articles must be clear, correct, and unambiguous. Any typographical or grammatical errors should be corrected at revision, so please note any specific errors here.

Reviewer #2: Yes

Reviewer #3: Yes

**********

6. Review Comments to the Author

Please use the space provided to explain your answers to the questions above. You may also include additional comments for the author, including concerns about dual publication, research ethics, or publication ethics. (Please upload your review as an attachment if it exceeds 20,000 characters)

Reviewer #2: Please present the 95% CIs the conventional way: Indicate both lower and upper limits of each interval in the tables and narrative.

Reviewer #3: (No Response)

**********

7. PLOS authors have the option to publish the peer review history of their article (what does this mean?). If published, this will include your full peer review and any attached files.

**Do you want your identity to be public for this peer review?** If you choose “no”, your identity will remain anonymous but your review may still be made public.

For information about this choice, including consent withdrawal, please see our Privacy Policy.

Reviewer #2: No

Reviewer #3: No

**********

[NOTE: If reviewer comments were submitted as an attachment file, they will be attached to this email and accessible via the submission site. Please log into your account, locate the manuscript record, and check for the action link "View Attachments". If this link does not appear, there are no attachment files.]

While revising your submission, please upload your figure files to the Preflight Analysis and Conversion Engine (PACE) digital diagnostic tool, https://pacev2.apexcovantage.com/. PACE helps ensure that figures meet PLOS requirements. To use PACE, you must first register as a user. Registration is free. Then, login and navigate to the UPLOAD tab, where you will find detailed instructions on how to use the tool. If you encounter any issues or have any questions when using PACE, please email PLOS at figures@plos.org. Please note that Supporting Information files do not need this step.

# Author comment: Author response to Decision Letter 2

**DOI:** 10.1371/journal.pgph.0000741.r006

20 Nov 2022

Attachment Submitted filename: Response to Reviewers.docx Click here for additional data file.

# Editor report: Decision Letter 3

**DOI:** 10.1371/journal.pgph.0000741.r007

**Authors:** Sabine Hermans

8 Dec 2022

Non-tuberculous mycobacterial pulmonary disease: Awareness survey of front-desk healthcare workers in Northern Tanzania

PGPH-D-21-00419R3

Dear Mr Maya,

We are pleased to inform you that your manuscript 'Non-tuberculous mycobacterial pulmonary disease: Awareness survey of front-desk healthcare workers in Northern Tanzania' has been provisionally accepted for publication in PLOS Global Public Health.

Before your manuscript can be formally accepted you will need to complete some formatting changes, which you will receive in a follow up email. A member of our team will be in touch with a set of requests.

Please note that your manuscript will not be scheduled for publication until you have made the required changes, so a swift response is appreciated.

IMPORTANT: The editorial review process is now complete. PLOS will only permit corrections to spelling, formatting or significant scientific errors from this point onwards. Requests for major changes, or any which affect the scientific understanding of your work, will cause delays to the publication date of your manuscript.

If your institution or institutions have a press office, please notify them about your upcoming paper to help maximize its impact. If they'll be preparing press materials, please inform our press team as soon as possible -- no later than 48 hours after receiving the formal acceptance. Your manuscript will remain under strict press embargo until 2 pm Eastern Time on the date of publication. For more information, please contact globalpubhealth@plos.org.

Thank you again for supporting Open Access publishing; we are looking forward to publishing your work in PLOS Global Public Health.

Best regards,

Sabine Hermans

Academic Editor

PLOS Global Public Health

***********************************************************

Reviewer Comments (if any, and for reference):
